# The Subcellular Localisation of the Human Papillomavirus (HPV) 16 E7 Protein in Cervical Cancer Cells and Its Perturbation by RNA Aptamers

**DOI:** 10.3390/v7072780

**Published:** 2015-06-26

**Authors:** Özlem Cesur, Clare Nicol, Helen Groves, Jamel Mankouri, George Eric Blair, Nicola J. Stonehouse

**Affiliations:** School of Molecular and Cellular Biology, Faculty of Biological Sciences and Astbury Centre for Structural Molecular Biology, University of Leeds, Leeds LS2 9JT, UK; E-Mails: bs09oc@leeds.ac.uk (Ö.C.); c.nicol@leeds.ac.uk (C.N.); h.groves14@imperial.ac.uk (H.G.); j.mankouri@leeds.ac.uk (J.M.); g.e.blair@leeds.ac.uk (G.E.B.)

**Keywords:** HPV16 E7, SELEX, RNA aptamers, cell surface localisation, ER retention

## Abstract

Human papillomavirus (HPV) is the most common viral infection of the reproductive tract, affecting both men and women. High-risk oncogenic types are responsible for almost 90% of anogenital and oropharyngeal cancers including cervical cancer. Some of the HPV “early” genes, particularly E6 and E7, are known to act as oncogenes that promote tumour growth and malignant transformation. Most notably, HPV-16 E7 interacts with the tumour suppressor protein pRb, promoting its degradation, leading to cell cycle dysregulation in infected cells. We have previously shown that an RNA aptamer (termed A2) selectively binds to HPV16 E7 and is able to induce apoptosis in HPV16-transformed cervical carcinoma cell lines (SiHa) through reduction of E7 levels. In this study, we investigated the effects of the A2 aptamer on E7 localisation in order to define its effects on E7 activity. We demonstrate for the first time that E7 localised to the plasma membrane. In addition, we show that A2 enhanced E7 localisation in the ER and that the A2-mediated reduction of E7 was not associated with proteasomal degradation. These data suggest that A2 perturbs normal E7 trafficking through promoting E7 ER retention.

## 1. Introduction

Papillomaviruses have been discovered in many vertebrates including humans, cattle, dogs, birds and reptiles. Within the papillomavirus family, around 170 human and 130 animal genotypes have been sequenced, identified and subsequently classified into 37 genera to date (see Papillomavirus Episteme (PaVE) [1]. Human papillomaviruses (HPVs) are classified into five genera (Alpha, Beta, Gamma, Mu and Nu) [2]. The Alpha genus contains high-risk mucosal HPVs that are the main cause of cervical cancer [3]. Amongst high-risk genotypes, HPV16 and 18 are most frequently found in cervical biopsies [4]. The majority (70%–90%) of HPV infections with both high- and low-risk genotypes are asymptomatic and can be cleared spontaneously within one to two years. Only a small percentage of persistent infections (around ~5%–10%) with high-risk types result in precancerous lesions. If untreated, these lesions may lead to squamous cell carcinoma (SCC) [5].

HPV-related anogenital and oropharyngeal cancers are a global health burden. Cancer Research UK reported around ~3000 new cases of cervical cancer in 2011 alone in the UK; the twelfth most common female cancer and third most common gynaecological cancer after uterine and ovarian cancers. In a worldwide perspective, cervical cancer is the second most prevalent cancer in women with an estimated 530,000 new cases in 2012 and around 270,000 deaths mostly in the developing world, according to the World Health Organisation (WHO) [6]. There is no HPV-specific cervical cancer treatment. However, protection can be achieved by the use of barrier methods and vaccination. Currently, there are two prophylactic vaccines in use based on virus-like particles (VLPs): the bivalent Cervarix (HPV16 and 18 VLPs) and the quadrivalent Gardasil (HPV6, 11, 16 and 18 VLPs). A nine-valent vaccine covering five additional high-risk HPVs is undergoing a Phase 3 clinical trial (trial number NCT00943722).

HPV encodes oncoproteins termed E5, E6 and E7. E7 is a highly phosphorylated, acidic polypeptide of approximately 100 amino acid residues. The E7 phosphoprotein has two conserved regions: CR1, CR2 and a carboxy-terminal region containing two zinc finger domains. CR2 contains a conserved LXCXE (L, leucine; C, cysteine; E, glutamate; X, any amino acid) motif, which is required for the interaction with pRb. E7 shares sequence similarities with the adenovirus (Ad) E1A protein and the simian vacuolating virus 40 (SV40) large tumour (large T) antigen [7]. Structural characterisation of HPV45 E7 [8,9] indicates that the N-terminus is intrinsically disordered while the C-terminus is highly structured with the zinc-binding region possibly involved in dimerisation. E7 has been shown to be present in dimeric form when expressed in *Escherichia coli* by gel filtration and non-denaturing acrylamide gel electrophoresis [10]. It has also been reported to form tetramers [11] and higher order oligomers [12] by analytical ultracentrifugation sedimentation equilibrium experiments.

The intracellular localisation of E7 has been investigated in detail. Subcellular fractionation experiments in CaSki cells demonstrated the presence of E7 in soluble cytoplasmic fractions [13]. Nuclear [14] and nucleolar [15] distribution in HPV16+ CaSki cells has also been proposed. Using antibodies with high discrimination capacity against monomeric, dimeric or oligomeric forms of E7, E7 dimers were shown to distribute to the nucleus, whilst oligomeric E7 displayed cytoplasmic distribution [16]. The presence of nuclear localisation and export sequences led to the hypothesis that E7 shuttles between the cytoplasm and nucleus [17]. Consistent with this, leptomycin B treatment has been shown to lead to E7 accumulation in the nucleus [17,18]. Cell confluency has also been proposed to dictate E7 localisation, being predominantly cytoplasmic in confluent cells but locating to both the nucleus and cytoplasm in sub-confluent cells [18], suggesting that location may be cell cycle-dependent.

Aptamers are short (15–100 nucleotides), single-stranded RNA or DNA molecules generated by SELEX (Systematic Evolution of Ligands by Exponential Enrichment), reviewed in [19,20,21]. Aptamers fold into specific complex structures that bind target proteins in a conformation-dependent manner and can interfere with function. Aptamers have been identified which recognise a number of viral proteins including HPV16 E6 and E7 [22,23,24], the RNA-dependent RNA polymerase of foot-and-mouth disease virus [25,26] and hepatitis C virus non-structural protein 5B [27,28]. An aptamer, targeted to VEGF termed pegaptanib (Macugen) was approved by the Food and Drug Administration (FDA) for the treatment of age-related macular degeneration in 2004 and examples of aptamers with anti-proliferative effects in cancer cells are currently undergoing clinical trials, including a guanosine-rich DNA oligonucleotide, AS1411 [29] and a L-RNA aptamer (Spiegelmer), NOX-12 [30].

We previously described an HPV16 E7 aptamer (termed A2) that resulted in a loss of E7 expression after transfection into HPV16+ cells [24]. We postulated that E7 was being targeted for degradation. Here, we show that aptamers can endocytose into early/late endosomes and that A2 redistributes E7 to the ER from the plasma membrane. We therefore propose that A2 interferes with normal E7 trafficking and cellular localisation.

## 2. Materials and Methods

### 2.1. Cell Culture

The SiHa cell line (ATCC No. HTB-35) was derived from a human squamous cell carcinoma of the cervix and contained 1–2 copies of the integrated HPV16 genome. CaSki cells (ATCC No. CRL-1550) were derived from a human epidermoid carcinoma of the cervix, and contain approximately 600 integrated copies. The HeLa cell line (ATCC No. CCL-2) was derived from a human adenocarcinoma of the cervix and contains approximately 10–50 copies of HPV18 genome. HaCaT cells are spontaneously-immortalised human keratinocytes (HPV negative). SaOS-2 (ATCC No. HTB-85) is an osteosarcoma cell line and negative for HPV DNA. SaOS-2, CaSki, SiHa, HeLa and HaCaT cells were maintained in DMEM containing 1% L-glutamine (GE Healthcare, Little Chalfont, Buckinghamshire, UK) supplemented with 10% foetal bovine serum (FBS) (PAA, Pasching, Austria), 100 units/mL penicillin (Lonza, Slough, UK), 0.1 mg/mL streptomycin (Lonza) in T-25 flasks. Flasks were maintained in a horizontal position in a humidified incubator (37 °C; 5% CO_2_). Cells were plated in 6-well (for protein extraction) or 12-well (for immunostaining) dishes for the experiments. In order to analyse the pathway of E7 degradation, CaSki cells were treated with a proteasome inhibitor, MG132 (Cayman Chemicals, Ann Arbor, MI, USA) at 100 µM for up to 6 h in the presence or absence of 100 nM aptamer.

### 2.2. Generation of 2′-Fluoro-Modified RNA Molecules

We have previously generated a library for templates of RNA aptamers selected against E7 [23]. A2 and SF1 aptamer templates were amplified by PCR and *in vitro* transcription was performed as previously described, incorporating 2′F U and C [22,23,24]. Aptamer 21-2 (5′-Cy5 and 3′-Cy3-labelled) and aptamer 47tr (5′-Cy3-labelled) both contained 2′F C and aptamer 21-2 also included 2′F U. 21-2 was purchased from Abgene (UK) and 47tr was synthesised by phosphoramidite chemistry in house [26,31].

### 2.3. Transfection of Cells with Aptamers Using Oligofectamine

Cells were transfected with aptamers at a final RNA concentrations of up to 100 nM using Oligofectamine (Invitrogen, Life Technologies, Waltham, MA, USA) according to the manufacturer’s instructions. 

### 2.4. Collection of Cells and Protein Extraction

Cells were lysed in radio-immunoprecipitation (RIPA) buffer [50 mM Tris-HCl (pH 8.0), 150 mM NaCl, 1% (*v*/*v*) Nonidet P-40, 0.5% (*w*/*v*) sodium deoxycholate, 0.1% (*w*/*v*) SDS] containing EDTA-free protease inhibitors (Roche, Penzberg, Germany) (1 tablet per 10 mL buffer), DNase I (5 µg/ mL) and 10 mM MgCl_2_ on ice for 30 min. Protein samples were mixed with 2 × Laemmli buffer and boiled to denature at 95 °C for 5 min.

### 2.5. SDS-PAGE and Western Blotting

This was performed as previously described [23]. Membranes were probed with rabbit anti-GAPDH (Sigma-Aldrich, St. Louis, MO, USA, used at 1:6000), mouse monoclonal HPV16 anti-E7; clone NM2 (Santa Cruz Biotechnology, Inc., Dallas, TX, USA, used at 1:200) overnight at 4 °C. Membranes were labelled with goat anti-mouse IgG peroxidase conjugate (Sigma, USA used at 1:2000) and goat anti-rabbit IgG peroxidase (Sigma, St. Louis, MO, USA, used at 1:1000) in 5% (*w*/*v*) milk for 1 h at room temperature.

### 2.6. Immunostaining of Cells and Fluorescence Microscopy

SiHa, SaOS-2, HeLa and HaCaT cells were grown on coverslips, washed with PBS and fixed with 4% (*v*/*v*) formaldehyde in PBS for 10 min at room temperature. Permeabilisation of cells was performed by incubation in 0.1% (*v*/*v*) TritonX-100/PBS for 10 min. Cells were incubated in blocking solution [1% BSA/Triton X-100 in 1 x PBS (*w*/*v*)] for 1 h at room temperature. Mouse monoclonal HPV16 anti-E7; clone 289–17013 (Abcam, UK used at 1:2000), mouse monoclonal HPV16 anti-E7; cloneNM2 (Santa Cruz Biotechnology, Inc., Dallas, TX, USA, used at 1:200), mouse monoclonal HPV18 anti-E7; clone 8E2 (Abcam, Cambridge, UK, used at 1:200), rabbit polyclonal anti-EEA1 (Millipore, Billerica, MA, USA, used at 1:500), rabbit polyclonal anti-LAMP-1 (CD107a) (Millipore, Billerica, MA USA used at 1:500) or rabbit polyclonal anti-LC3 (Abcam, Cambridge, UK used at 1:2000) were added to cells and incubated overnight at 4 °C on a rotating platform. To analyse cell-surface staining, cells were incubated with anti-E7 at 4 °C prior to fixation for 1 h.

Cells were washed with PBS and incubated with fluorochrome-conjugated secondary antibodies AlexaFluor 568 F goat anti-mouse IgG (H+L), AlexaFluor 488 chicken anti-mouse IgG (H+L), AlexaFluor 488 goat anti-rabbit IgG (H+L). Secondary antibodies (all from Life Technologies, Waltham, MA, USA) were used at 1:500 in 1% BSA/Triton X-100 for 2 h at room temperature and kept in the dark. Glass coverslips were mounted on microscope slides in Vectashield-DAPI stain (Vector laboratories, Burlingame, CA, USA) and viewed on Zeiss LSM 510 upright or LSM 700 inverted confocal microscopes.

### 2.7. Quantitation of Co-Localisation by Imeris Software

Confocal images were analysed by Bitplane:Imeris image analysis software in order to calculate the Pearson’s co-localisation coefficient values. Results are presented as % of co-localisation between two different channels and n refers to the number of cells analysed.

## 3. Results

### 3.1. A2-Mediated Degradation of E7 Is Not Mediated via Proteasomal Pathways

**Figure 1:**
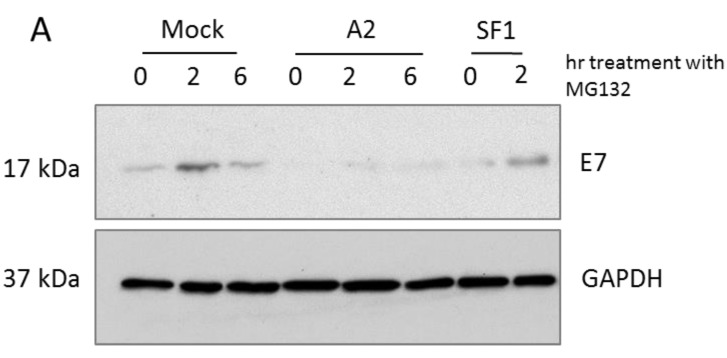

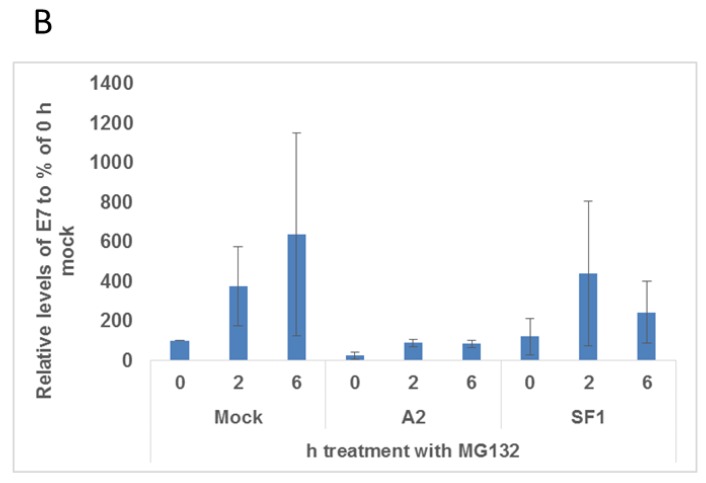
Proteasomal degradation of E7 was not affected by A2 treatment. CaSki cells were mock-transfected or transfected with 100 nM A2 or SF1 for 16 h. Cells were treated with MG132 (100 µM) for the indicated time points and immunoblot analysis of cell lysates was performed to detect E7 and GAPDH (as a housekeeping control) (**A**); Mean data from three independent experiments is shown, together with standard errors (**B**).

We have previously described a loss of E7 in A2-transfected HPV16+ CaSki cells. The effect was not observed with a control aptamer SF1, selected to an unrelated protein [24]. Here, the peptide-aldehyde proteasome inhibitor, MG132 (carbobenzoxyl-L-leucyl-L-leucyl-L-leucinal) was used to study E7 degradation in the presence or absence of A2. CaSki cells transfected with A2 or a control aptamer (SF1) for 14–16 h were treated with 100 µM MG132 for up to 6 h. Figure 1 demonstrates that transfection of the A2 aptamer appeared to result in a lower level of E7, as evidenced by the level at t = 0 (corresponding to 14–16 h post-transfection), in agreement with previous data [24]. Furthermore, in A2-transfected cells, E7 levels remained low. MG132 treatment clearly had little effect, indicating that newly-synthesised E7 was still being degraded. In contrast, the level of MG132-mediated inhibition appeared to be highly variable in the mock- or SF1-treated cells and E7 did appear to accumulate, suggesting that the protein normally undergoes proteasomal degradation. The clear differences between the E7 levels in the mock- or SF1-treated cells compared to A2-treated cells led to the suggestion that A2 acts to enhance E7 degradation by a non-proteasomal mechanism.

### 3.2. E7 Localises to the Plasma Membrane

**Figure 2:**
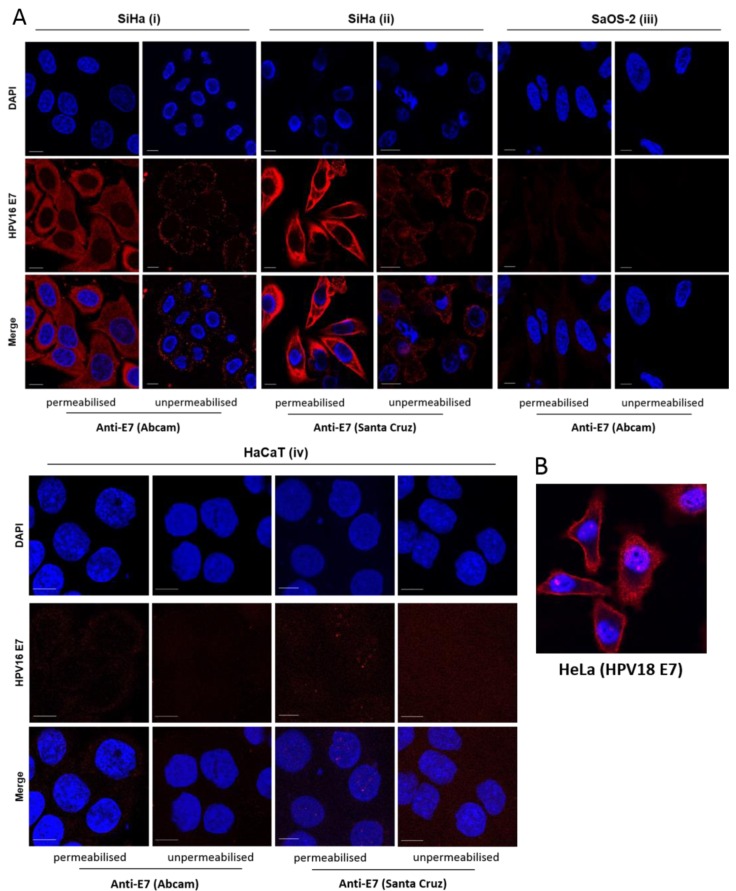
E7 distributes to the plasma membrane of HPV-transformed cells. (**A**) HPV16 E7+ SiHa cells (i and ii) and SaOS-2 cells (iii) HaCaT cells (iv) (as negative controls, containing no E7) were fixed, permeabilised and incubated with antibodies recognising full-length HPV16 E7 (i) and (iii) and residues 35–56 (ii). A comparable group of live cells was incubated with these antibodies in parallel. Cells were fixed and stained with AlexaFluor568 anti-mouse antibodies. Fluorescent imaging was performed using either a Zeiss LSM700 or LSM510 inverted microscope. The scale bar = 10 µm. Representative images are shown; (**B**) HeLa cells were fixed, permeabilised and labelled with anti-HPV18 E7 antibodies, which recognise residues 36–70, followed by staining with AlexaFluor594 goat anti-mouse IgG.

**Figure 3:**
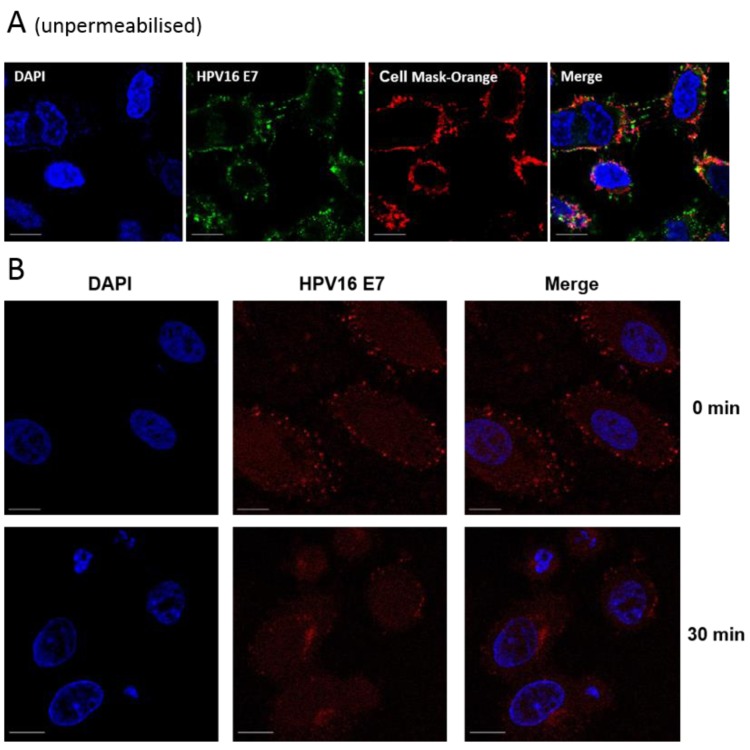
(**A**) Co-distribution of E7 with the cell membrane marker CellMask Orange (red) in unpermeabilised SiHa cells. Live cells were dual-stained with anti-E7 (Abcam) and CellMask Orange; (**B**) Time and temperature-dependent internalisation of cell surface E7, chased by anti-E7 antibody (Abcam). SiHa cells were incubated with the anti-E7 antibody for 1 h at 4 °C and incubated at 37 °C for 30 min to allow E7 internalisation. Cells were fixed, permeabilised and stained with AlexaFluor568 anti-mouse antibodies. Fluorescent imaging was performed using either a Zeiss LSM700 or LSM510 inverted microscope. The scale bar = 10 µm.

We next sought to investigate the intracellular distribution of E7. Using HPV16 E7-specific antibodies in HPV16+ SiHa cells, we observed that E7 displayed diffuse cytoplasmic staining, as expected, however this appeared to extend to the cell periphery, characteristic of localisation to the plasma membrane or plasma membrane-associated sub-membrane cisternae (Figure 2A). It is interesting to note that HeLa cells (HPV18+) show a similar distribution of E7, Figure 2B. We aimed to confirm this observation by detecting E7 staining in unpermeabilised SiHa cells. These experiments (with two different anti-HPV16 antibodies) demonstrated E7 localisation at the cell surface (Figure 2A, i and ii). No such pattern of staining was evident in SaOS-2 cells or HaCaT cells (HPV-negative cell lines), confirming the staining to be E7 specific (Figure 2A, iii and iv). We also employed a plasma membrane-specific stain (CellMask Orange). This stain is an amphipathic molecule, consisting of a lipophilic region and a negatively charged hydrophilic dye for membrane loading and the attachment of the probe in the plasma membrane, respectively [32,33]. Use of CellMask also demonstrated the presence of E7 at the plasma membrane, Figure 3A.

It should be noted that we were unable to produce membrane fractions that were completely free of cytoplasmic contamination, therefore were unable to confirm E7 localisation by cell fractionation (data not shown). However, we investigated the ability of E7 to undergo internalisation and removal from the plasma membrane, using protocols documented for the study of other cell-surface proteins [34]. Cells were maintained at 4 °C to allow antibody attachment followed by incubation at 37 °C for 30 min to follow the internalisation of cell-surface E7. Redistribution of E7, from a peripheral cell surface localisation (observed at *t* = 0) to the peri-nuclear region of the cells (at *t* = 30 min) was observed. This strongly suggested that E7 is endocytosed from the plasma membrane to an intracellular compartment, indicative of rapid internalisation from the cell surface (Figure 3B).

### 3.3. Aptamers Localise to Early/Late Endosomes upon Transfection

The internalisation of ligands, extracellular molecules, plasma membrane proteins and lipids commonly occur by endocytosis. Typically, endocytosed cargo is delivered to early endosomal compartments, followed by transit to the late endosomes/lysosomes for degradation, to the trans-Golgi network (TGN) or recycling endosomes in order to return the cargo back to the plasma membrane, reviewed in [35]. However, little is known about the sub-cellular localisation of RNA molecules after uptake.

**Figure 4:**
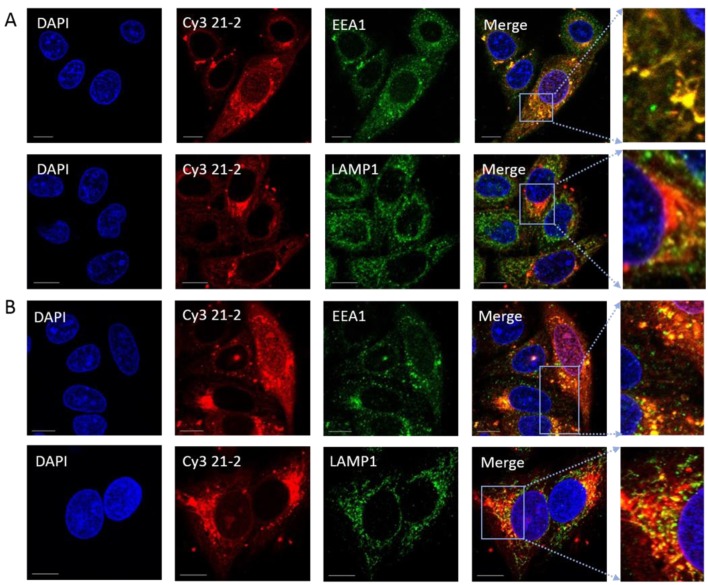

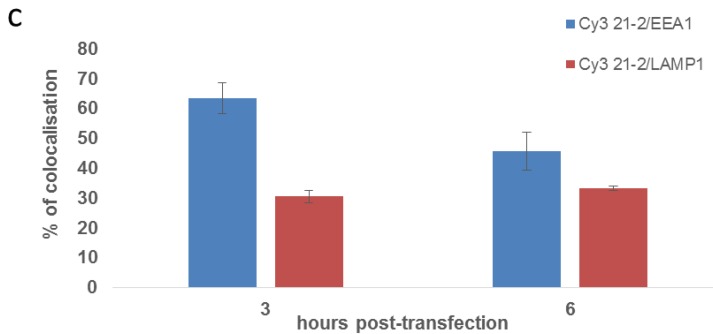
A model aptamer (aptamer 21-2) localises predominantly in early and late endosomes in SiHa cells following Oligofectamine transfection. Cells were transfected with 80 nM Cy3-labelled 21-2 using Oligofectamine. At 3 (Panel (**A**)) or 6 h (Panel (**B**)) post-transfection, cells were fixed, permeabilised and incubated with primary antibodies anti-EEA1 or anti-LAMP1 (staining early and late endosomes, respectively) prior to incubation with AlexaFluor488 goat anti-rabbit secondary antibody. The right hand side panel is a zoom of merged images. The scale bar = 10 µm. The level of co-localisation was analysed by Bitplane:Imeris image analysis software (Panel (**C**)). For EEA1 and LAMP1 colocalisation respectively, *n* = 8 and 29 at 3 h and *n* = 30 and 19 at 6 h. Red, green and blue are Cy3 (21-2), FITC (early or late endosomes) and DAPI (nucleus), respectively.

To investigate uptake of RNA aptamers in E7-expressing cells, we utilised chemically-synthesised, model aptamers (*i.e.*, not selected to bind to E7), labelled with Cy3 [26,31] and assessed their transit into cellular compartments at defined time points post-incubation. Counting the number of aptamer-postive cells revealed a transfection efficiency of 87% (*n* = 90). At 3 h post-transfection, co-localisation with EEA1 demonstrated that the cy3-labelled aptamer 21-2 predominantly localised to early endosomes (63.3%, Figure 4A,C). However, in cells transfected for 6 h, this co-localisation was reduced and the aptamer was localised to both early and late endosomes/lysosomes (45.5% and 33.2% respectively, Figure 4B,C). A second aptamer, Cy3-labelled 47tr, displayed a similar pattern of distribution to Cy3 21-2 in SiHa cells (data not shown). Taken together these data suggest that RNA aptamers are targeted to early/late endosomal/lysosomal pathways following cell entry. However, the fate of these aptamers at later time points is currently unknown.

### 3.4. Localisation of E7 in Cellular Compartments upon Aptamer Transfection

We have previously shown that aptamer A2 can reduce E7 levels [24] but the mechanism of this effect was not addressed. Given our finding that E7 can distribute to the plasma membrane (Figure 2 and Figure 3) we hypothesised that the A2 aptamer may bind cell surface E7, and mediate E7 degradation via the endosomal/lysosomal pathways. As labelling of A2 may have effects on conformation and therefore function, indirect methods were used to evaluate the effects of A2 on E7. Co-localisation studies with E7 and endosomal markers were performed to assess the cellular distribution of E7 in the presence or absence of A2 and the control aptamer, SF1. Cells were fixed 16 h post-transfection, in order to allow time for any A2-mediated effects to become apparent [24]. It should be noted that we were unable to quantitate transfection efficiency with A2 or SF1. These aptamers are similar in size, but somewhat larger than 21-2. It is therefore likely that the transfection will be less efficient.

**Figure 5:**
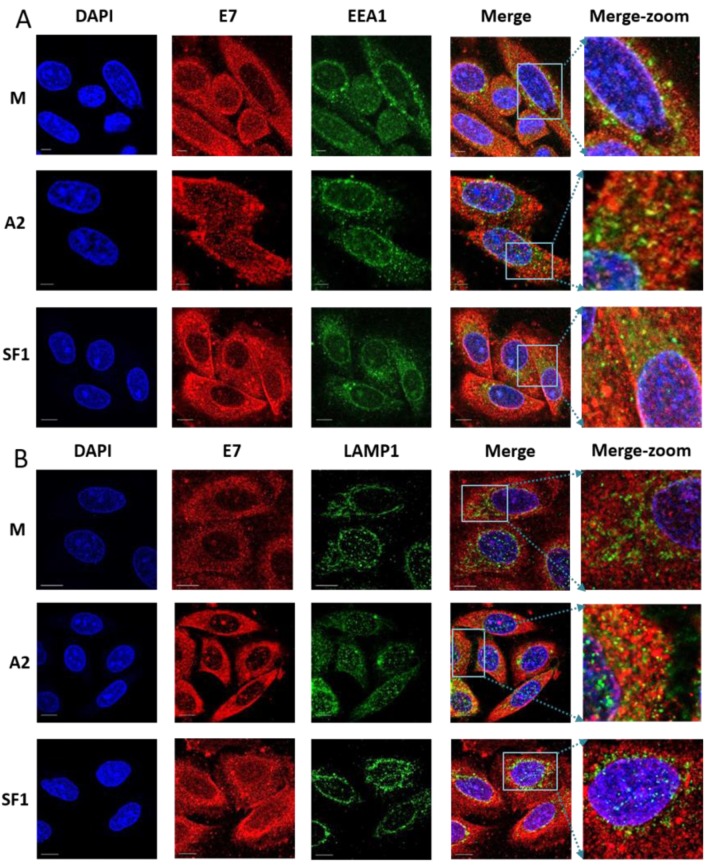

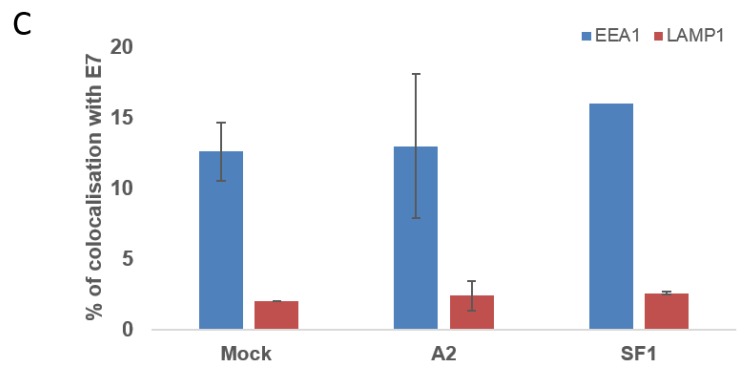
E7 does not co-localise with the early endosomal marker, EEA1 (**A**) or the late endosomal (lysosomal) marker, LAMP1 (**B**). SiHa cells were mock-transfected (M) or transfected with 100 nM A2 or SF1 using Oligofectamine. At 14 h post-transfection, cells were co-stained with either anti-EEA1 or anti-LAMP1 (staining early and late endosomes, respectively) prior to incubation with AlexaFluor488 goat anti-rabbit secondary antibody. Cells were then incubated with anti-E7 antibody (Abcam) followed by AlexaFluor568 rabbit anti-mouse secondary antibody. Red, green and blue are E7, FITC (early or late endosomes) and DAPI (nucleus), respectively. The last panel is a zoom of merged images. The scale bar = 10 µm. E7/EEA1 and E7/LAMP1 colocalisation were analysed using Bitplane:Imeris image analysis software based on the calculation of Pearson’s correlation coefficient values and presented as percentage co-localisation (**C**). *n* = 27, 23 and 4 for E7/EEA1 and *n* = 12, 24 and 9 for E7/LAMP-1 in mock-, A2- and SFI-treated SiHa cells, respectively.

**Figure 6:**
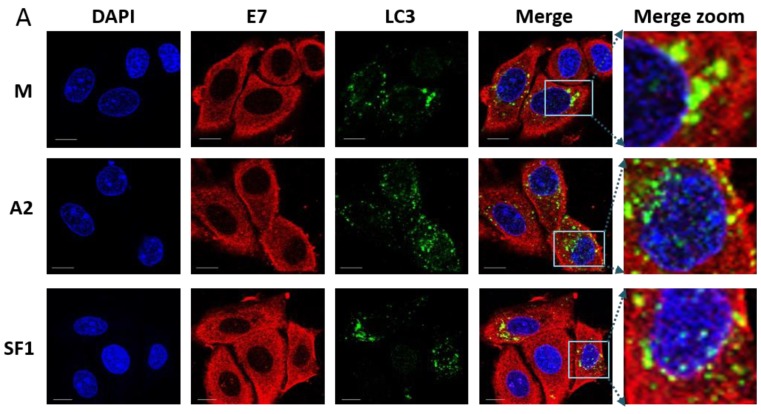

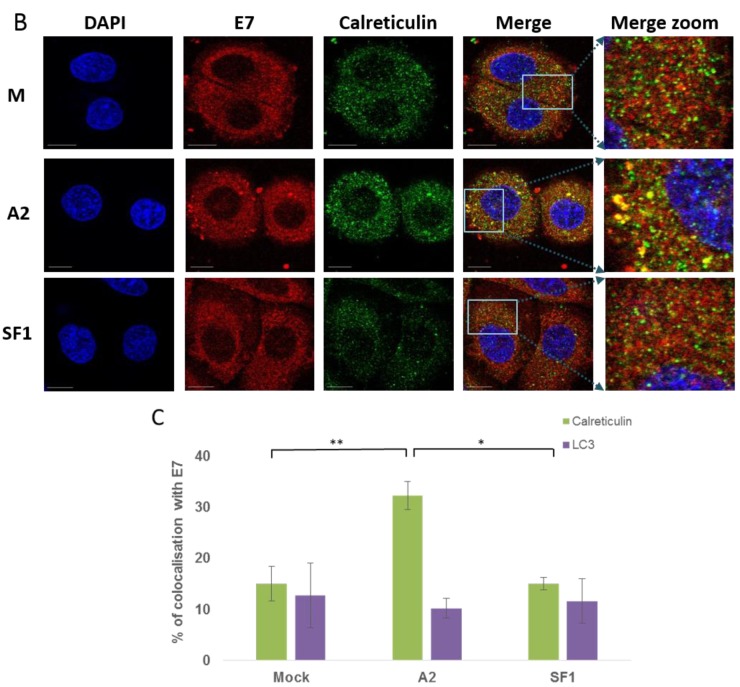
E7 does not co-localise with an autophagosome marker, LC3 (**A**) but appears to co-localise with endoplasmic reticulum marker calreticulin (**B**) in the presence of A2. SiHa cells were mock-transfected (M) or transfected with 100 nM A2 or SF1 using Oligofectamine. At 14 h post-transfection, cells were co-stained with anti-E7 (Abcam) and either anti-LC3 (an autophagosomal marker) or anti-calreticulin (an ER marker) prior to incubation with either AlexaFluor568 rabbit anti-mouse or AlexaFluor488 goat anti-rabbit secondary antibodies. Red, Green and Blue are E7, FITC (LC3, autophagosome) and DAPI (nucleus), respectively. The last panel is a zoom of merged images. The scale bar = 10 µm. E7/LC3 and E7/calreticulin colocalisation was analysed using Bitplane:Imeris image analysis software based on the calculation of Pearson’s correlation coefficient values and presented as percentage co-localisation (**C**). *n* = 21, 23 and 17 for E7/LC3 and *n* = 19, 39 and 20 for E7/calreticulin with mock-, A2- and SFI-treatment, respectively. * *p* ≤ 0.0005, ** *p* ≤ 0.002.

We observed only minimal co-localisation of E7 with the endosomal marker EEA1 (Figure 5A,C) with values of 12.6%, 13.0% and 16.0% for mock-, A2- and SF1-treated SiHa cells, respectively. There were no statistical significant differences between the three treatments. It should be noted that these data were collected at a later timepoint than the data shown in Figure 3, as we aimed to allow time for A2-mediated effects on E7 to become apparent. It is therefore possible that co-localisation between E7 and endosomal markers may have been evident at earlier times post-transfection. Co-localisation with LAMP1 (Figure 5B,C) was low and very similar for the three treatments (ranged between 2.0% and 2.5%, not significant. The level of colocalisation between the autophagosomal marker LC3 (Figure 6A,C) and E7 also showed no difference between the three treatments (12.7%, 10.2%, 11.6%). In contrast, there was an increased co-localisation of E7 with the ER marker, calreticulin, upon transfection with A2 (Figure 6B,C). Co-localisation in mock- and SF1-treated cells was 15.1% and 15.1%, but significantly higher at 32.3% in A2-transfected cells (*p* ≤ 0.01). Taken together, these data suggest that A2 perturbs normal E7 trafficking and cellular distribution through enhancing E7 ER retention following E7 binding.

## 4. Discussion

This work is the first to describe the localisation of E7 to the plasma membrane and to propose a mechanism for E7 degradation in the presence of the E7-specific aptamer, A2. Three independent antibodies recognising full-length HPV16 E7, HPV16 E7 residues 35–56 and HPV18 E7 residues 36–70 were employed to show that E7 distributed to the plasma membrane. It is therefore likely that the N-terminal region of E7 is accessible and extracellularly-exposed since we could detect E7 in unpermeabilised SiHa cells. We were unable to produce membrane fractions that were completely free of cytoplasmic contamination, therefore were unable to confirm E7 localisation by cell fractionation. However, the data above, together with the ability of E7 to undergo internalisation and removal from the plasma membrane provide compelling evidence for membrane localisation. It should be noted that the amount of membrane-associated E7 is relatively low and this could explain why this has not been detected previously [13,36]. The level of cell confluency could also be a factor [18]. Although the data with HPV18 is preliminary, it is possible that this effect may be common across other HPV types. Further work would be necessary in order to establish whether this is the case.

We have previously reported that transfection of CaSki cells with the aptamer A2 resulted in a decrease of E7 levels with an accompanying increase in pRb, which was suggested as the reason behind the apoptotic effects of A2 observed in HPV-transformed cells [24]. The loss of E7 in A2-transfected cells was previously suggested to be due to the inability of A2-bound E7 to interact with its major cellular partners including pRb, thus remaining unfolded which could lead to destabilisation and degradation [24]. The proteasome/ubiquitin system is primarily responsible for clearance of cellular misfolded/unfolded proteins. E7 is a short-lived protein with a half-life of 30–40 min [13], allowing detection of protein accumulation within a few hours of addition of MG132. Consistent with previous studies [37], we showed that degradation of E7 can be mediated by 26S proteasomes as MG132 treatment resulted in the accumulation of E7. However, in the presence A2, E7 accumulation was much less evident. Given this data, we propose an alternative non-proteasomal mechanism for aptamer-mediated E7 degradation.

**Figure 7:**
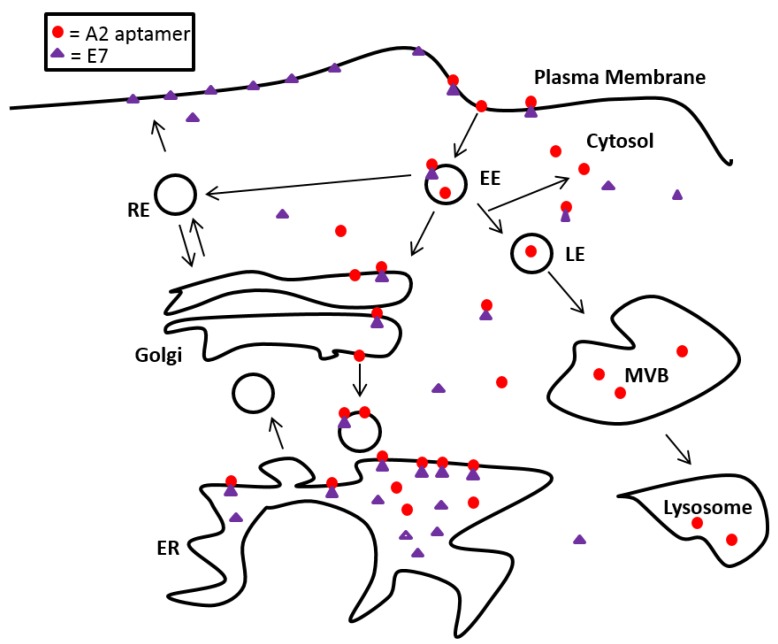
A2 can enter the cells alone or via the endosomal system. Upon contact with E7; either at the plasma membrane or intracellularly, A2 enhances E7 retention in the ER. This would be predicted to reduce E7 delivery to the plasma membrane via its normal biosynthetic route. ER: endoplasmic reticulum, EE: early endosome, LE: late endosome, MVB: multivesicular bodies, RE: recycling endosome.

Studies with a control, labelled aptamer revealed that this molecule localised to both early and late endosomes. Localisation to early endosomes was particularly striking at early timepoints post-transfection (3 h). It is interesting to note that the reduction in co-localisation seen after 6 h did not correlate with an increase in co-localisation with the late endosomal marker. Further time points might be useful, however, this could be indicative of escape of aptamers from the endocytic pathway. Whilst there is a paucity of data on the sub-cellular localisation of aptamers, transfection of siRNAs using cationic lipids, nanoparticles or cell-type-specific delivery reagents have shown that siRNAs are trafficked to the endosomal pathway (early and then late endosomes), reviewed in [38]. It is clear that such siRNA molecules must escape the endosomal pathway to function, and the observation led to a hypothesis for A2-mediated activity. E7 on the cell surface could be internalised along with the bound aptamer during transfection, resulting in its accumulation in endosomes and then late endosomes/lysosomes for degradation. In order to investigate this hypothesis, HPV16+ SiHa cells were co-stained with E7 and several endosomal markers in the presence and absence of A2. We found no evidence of E7 accumulation in lysosomes or autophagosomes in aptamer-transfected or mock-transfected SiHa cells after 14 h. However, E7 localisation in the ER (using calreticulin as a marker) increased in cells transfected with A2. Interestingly, E7 localisation to the ER has been demonstrated previously in CaSki cells (using calnexin as a marker) [39]. These authors also noted localisation to the Golgi, we have not investigated whether A2 has any effect here. Our data suggest that a possible conformational change of E7, mediated by A2 binding, leads to an accumulation in the ER. This would be predicted to perturb normal biosynthetic delivery of E7 to the plasma membrane or other organelles (Figure 7). As E7 does not possess common peptide ER retention motifs such as [K/H]DEL, KKXXX, KXKXXX and PL[Y/F][F/Y]XXN, it is likely that A2 binding prevents correct E7 folding/oligomerisationleading to its accumulation in an unfolded/aberrant state. This accumulation of unfolded E7 and subsequent stimulation of related stress responses may contribute to the stimulation of apoptosis previously observed in HPV+ cells [24,40,41,42]. Future work (including the use of specific inhibitors) would be necessary to validate this model.

## 5. Conclusions

In summary, the data reported here demonstrate the utility of RNA aptamers as molecular tools to aid our understanding of the cellular function of the HPV E7 oncoprotein.
